# Supplementary figures and images for: Identification of antimicrobial peptides from the Ambystoma mexicanum displaying antibacterial and antitumor activity
Source: PLoS One. 2025 Mar 5;20(3):e0316257. doi: 10.1371/journal.pone.0316257 (PMC11882074; doi:10.1371/journal.pone.0316257)

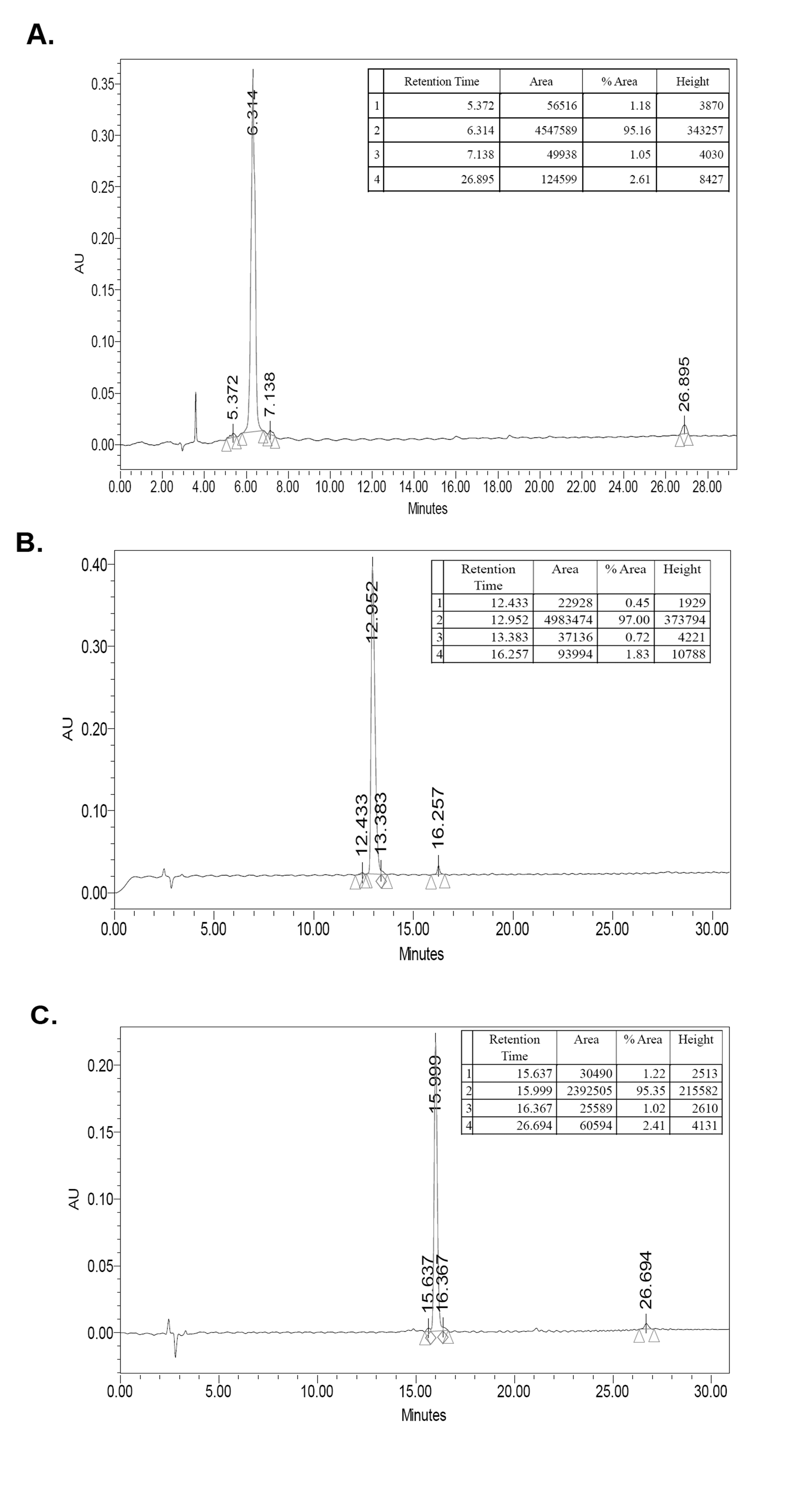

Supplement: S1 Fig — (TIF) [file pone.0316257.s001.tif]

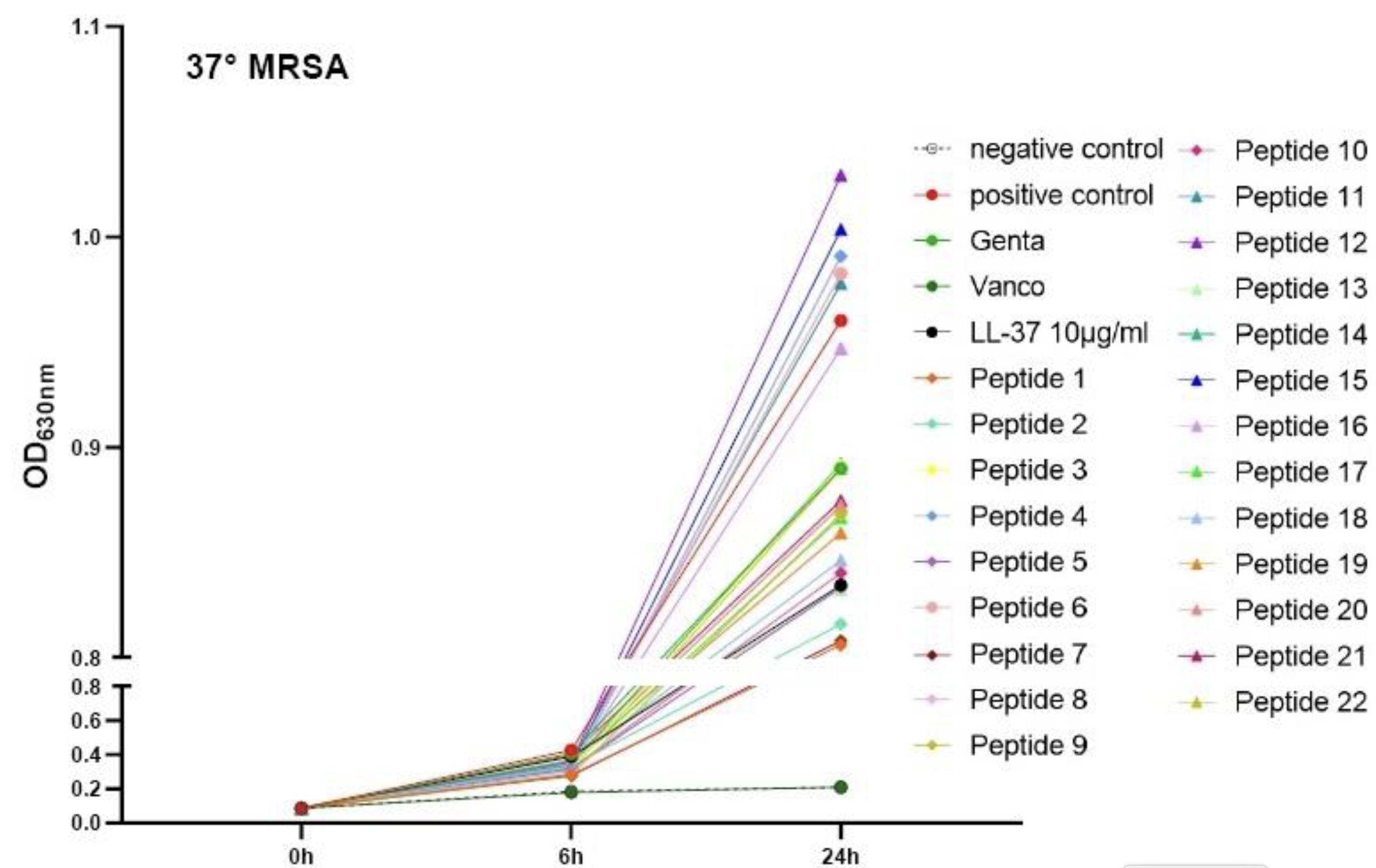

Supplement: S2 Fig — (PNG) [file pone.0316257.s002.png]

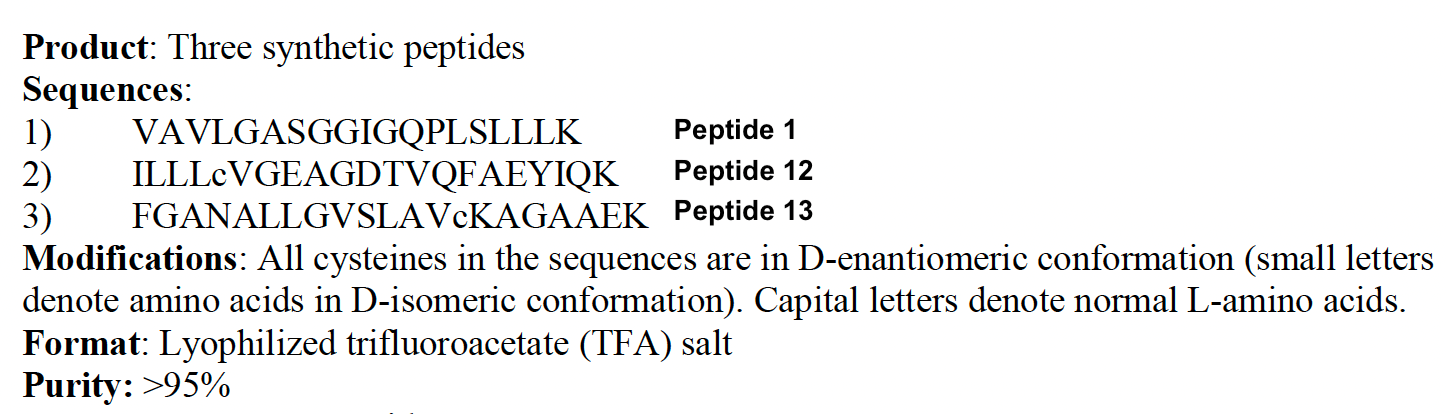

Supplement: S3 Fig — Peptide 1 (VAVLGASGGIGQPLSLLLK), Peptide 12 (ILLLcVGEAGDTVQFAEYIQK), and Peptide 13 (FGANALLGVSLAVcKAGAAEK). (TIF) [file pone.0316257.s003.tif]
