# Supplementary material for: Identification of antimicrobial peptides from the Ambystoma mexicanum displaying antibacterial and antitumor activity
Source: PLoS One. 2025 Mar 5;20(3):e0316257. doi: 10.1371/journal.pone.0316257 (PMC11882074; doi:10.1371/journal.pone.0316257)
Supplement: Attachment — Submitted filename: Comments to the authors_PLOSOne_2024.docx [file pone.0316257.s004.docx]

PLOSOne 2024

Comments to the authors

The manuscript presents original research on the identification and characterization of antimicrobial peptides (AMPs) derived from the skin mucus of *Ambystoma mexicanum* (axolotl). While the study has the potential to contribute valuable insights to the field, several critical aspects need to be addressed to meet the high technical standards and publication criteria of PLOS ONE.

**Evaluation Based on PLOS ONE Criteria**

1. **Original Research:** **Partially Meets Criteria:** The study contributes original findings regarding AMPs. However, the depth and novelty are somewhat undermined by the lack of detailed methodological justifications and comprehensive data presentation.
2. **Results Not Published Elsewhere:** The manuscript does not indicate prior publication of the results.
3. **High Technical Standard and Detailed Description:**

**Does Not Adequately Meet Criteria:**

- **Collection and Permissions:** The manuscript does not specify the origin of the axolotls or the collection permits. On page 5, line 110, it is mentioned that the axolotls were bred and kept at the AMBC. However, the manuscript should clarify where and how they were originally collected. What was the collection location, and under what license or collection permit were they obtained? This information should be indicated. Include information on the collection of axolotls and relevant permits.
- **Chromatography and Mass Spectrometric Analysis Details:** The equipment used (brand and model) and the injection volumes are not specified. Page 6, line 125. Authors should specify the chromatography equipment used, injection volumes, and detailed peptide identification methods.
- **Peptide Identification:** Insufficient details are provided about the identification of the peptide sequences and the bioinformatics tools used and comparisons with existing databases. Page 6, lines 130-133. The authors should attach supplementary information including the spectra of the 22 selected peptides (or at least some examples) from which they identified the peptide sequences by MSMS. Authors should provide comprehensive information on the bioinformatics tools and comparisons with existing databases. Indicate how similar the identified sequences are to those in the databases. Are they novel sequences? What percentage of similarity do they have with those already described for other species? Table 1. In table 1 there are 3 peptides highlighted in yellow and neither the text nor the table indicates why they are highlighted.
- **Antimicrobial Assays:** The final concentration of the inoculum and the controls for the assays are not indicated. Page 7. Authors should indicate the MIC values in µg/ml and µM. The results are very good, in the discussion the authors should compare the MIC (activity) results with those obtained for other peptides already described in other species against the same resistant strains or compare with current antibiotics and compare their potential.
- **Cell Cultures:** The source of the RPMI-1640 medium and fetal calf serum is not provided.
- **Apoptosis Assay:** The number of replicates and the use of a positive control are not mentioned. The value obtained for the negative control is not shown (Page 7 and Figure 2). Figure 2: The figure should include the results of both the negative control (cells treated with the vehicle, without any peptide) and the positive control (cells treated with a known apoptosis-inducing agent, such as a chemotherapeutic drug or a known caspase inducer). The absence of a positive control makes it difficult to compare the apoptotic potency of the peptides with a known standard. The authors should provide a detailed explanation of why a positive control was not used and how this impacts the validity of the results.
- **PCR:** The forward and reverse primers used are not mentioned (page 8, line 183).
- **Data Availability:** There is no table or supporting information on the identified sequences, nor is their availability in public databases indicated. Authors should include a table with the identified peptide sequences and ensure their availability in public databases.

4. **Data Supporting Conclusions:**

**Partially Meets Criteria:** While the data appears to support conclusions regarding the antimicrobial and apoptotic activity of certain peptides, the lack of complete statistical analyses and insufficient data presentation weakens the robustness of these conclusions.

5. **Intelligible Presentation and Standard English:**

**Meets Criteria:** There are no significant language issues reported, although the clarity of data presentation could be improved.

6. **Other details**

INTRODUCTION

Clarify what MRSA means because it is introduced for the first time.

MATERIALS AND METHODS

Page 7. Line 142 clarify MSSA because it is being introduced for the first time.

Page 7. Line 146 and 160 please correct CO_2_

Page . 7 line 148. the word "representing" should be omitted.

Page 7 line 157 missing the source of FCS

Page 7 line 155, 164, Figure 2B, and the rest of the document, unify: T-47D/T47D/T47-D

Page 7, line 157, 164 and the rest of the document unify: MCF 10A/MCF10A

Page 8, line 179. Correct "(REF 740955.50)" is missing reference.

Page 8, line 186. It does not make sense to use the abbreviation ACTB because it is not used throughout the work.

Page 8, line 187. Cite the ΔΔCT method.

RESULTS

Page 9, line 200-201 Delete the sentence "These peptides were synthetically produced by the commercial supplier Caslo Laboratory, Denmark" already indicated in materials and methods.

Authors should add a table with the activity results, indicating the MIC values in µg/ml and µM

Page 9 212-217. Figure 2 analysis: According to the analysis of the figure, the statistical comparison is performed between different cell types rather than among the peptide concentrations. Consequently, it is not possible to conclude that the effects on apoptosis are greater at higher concentrations. The authors report that peptides 1, 12, and 13 exhibited the highest induction of apoptosis. However, results for the other 19 peptides are not shown. Additionally, while peptides 1 and 12 are reported to induce apoptosis at low concentrations, no significant difference analysis is observed compared to peptide 13. The authors should address these issues by providing a more detailed statistical analysis and including data for all tested peptides to better support their conclusions.

Page 10, line 217. It mentions that peptides 1 and 12 increased apoptosis at a concentration of 1µg, however in materials and methods it says that they tested concentrations 2.5, 5 and 10µg/ml. Please clarify which is incorrect. And in any case, also correct figure 2.

DISCUSION

The results are very good, in the discussion the authors should compare the MIC (activity) results with those obtained for other peptides already described in other species against the same resistant strains or compare with current antibiotics and compare their potential.

Addressing these points will significantly enhance the manuscript's alignment with PLOS ONE's criteria and increase its likelihood of acceptance for publication.
