# Supplementary material for: Identification of antimicrobial peptides from the Ambystoma mexicanum displaying antibacterial and antitumor activity
Source: PLoS One. 2025 Mar 5;20(3):e0316257. doi: 10.1371/journal.pone.0316257 (PMC11882074; doi:10.1371/journal.pone.0316257)
Supplement: Attachment — Submitted filename: response to reviewers.docx [file pone.0316257.s006.docx]

**Reviewer 1#** Thank you for your positive feedback and recommendation for publication without changes. We appreciate your recognition of our project design and outcomes, and we are encouraged by your support for further research in the area of antimicrobial peptide activity.

**Reviewer 2#**

1. We understand your concern about how massage might affect AMP expression in axolotl skin mucus.  However, we respectfully assert that the massage technique does not significantly alter the mucus composition, but rather increases the quantity produced. This method is well-established and non-invasive, primarily stimulating the release of pre-existing secretions stored in granular glands beneath the skin^1,2^. Collecting "normally produced" mucus without stimulation would yield insufficient quantities for comprehensive analysis, potentially underrepresenting less abundant peptides.
   1) Demori, I., El Rashed, Z., Corradino, V., Catalano, A., Rovegno, L., Queirolo, L., ... & Grasselli, E. (2019). Peptides for skin protection and healing in amphibians. *Molecules*, *24*(2), 347.
   2) O'Rourke, D. P. (2007). Amphibians used in research and teaching. *ILAR journal*, *48*(3), 183-187.
1. Thank you for your valuable feedback on our peptide identification process from the collected mucus. To clarify, we utilized an AXIMA Performance MALDI-TOF/TOF mass spectrometer (Shimadzu) at the Institute for Toxicology in Hannover for this purpose. The chromatography fractions were analyzed directly by this mass spectrometer, and peptide identification was performed using the instrument's integrated software, which matches experimental spectra to theoretical sequences in protein databases. We have revised the manuscript to include these details for greater clarity and reproducibility of our methods. Thank you for bringing this to our attention.
2. We used the CAMP3 (Collection of Anti-Microbial Peptides) database and its classifiers (SVM, RF, ANN, and DA) to predict the antimicrobial potential of the identified peptides. We focused on peptides that showed high probability of antimicrobial activity across multiple classifiers. The final 22 peptides were selected based on their consistently high predicted antimicrobial activity and other promising bioactive properties. We have revised our manuscript to include more specific details about the selection criteria in this process. For basic properties see table 1.
3. 4. We have included the sequences of three peptides, these are the ones that show significant antimicrobial activity, in Supplementary Table S3.
5. We did not treat the bacteria first with the crude extract and then with each of the peptides. Our antimicrobial activity tests only involved treating the bacteria with individual synthesized peptides.
6. For the antimicrobial tests, we used a range of concentrations (0.5 to 100 μg/mL) based on standard protocols from CLSI [1] and previous studies on similar antimicrobial peptides [2]. This range allowed us to determine the minimum inhibitory concentration (MIC) and observe dose-dependent effects. For the antitumor activity tests, we selected concentrations (0.1 to 50 μM) based on preliminary experiments and literature data on related peptides [3], ensuring we could calculate IC50 values while staying within solubility limits and below highly cytotoxic levels [4,5].
1) Clinical and Laboratory Standards Institute. Methods for Dilution Antimicrobial Susceptibility Tests for Bacteria That Grow Aerobically; Approved Standard—Tenth Edition. CLSI document M07-A10. Wayne, PA: CLSI; 2015.
2) REW, Sahl HG. Antimicrobial and host-defense peptides as new anti-infective therapeutic strategies. Nat Biotechnol. 2006;24(12):1551-1557.
3) Mahlapuu M, Håkansson J, Ringstad L, Björn C. Antimicrobial Peptides: An Emerging Category of Therapeutic Agents. Front Cell Infect Microbiol. 2016;6:194.
4) Hoskin DW, Ramamoorthy A. Studies on anticancer activities of antimicrobial peptides. Biochim Biophys Acta. 2008;1778(2):357-375.
5) Mader JS, Hoskin DW. Cationic antimicrobial peptides as novel cytotoxic agents for cancer treatment. Expert Opin Investig Drugs. 2006;15(8):933-946.
7. In future experiments, we will incorporate hemolysis tests to assess the potential toxicity of our antimicrobial peptides to red blood cells. This will allow us to evaluate the therapeutic index of the peptides, ensuring that we identify candidates with strong anticancer activity while minimizing hemolytic effects.
8. We have performed appropriate statistical tests to demonstrate the significance of differences observed between controls and treatments. Specifically, we used student -t test to compare the two groups. The results of these tests have been added to the manuscript (Figure2), including p-values for significant differences.
9. We respectfully maintain that our OD-based kinetic graphs effectively illustrate antimicrobial activity. While % growth offers standardization, OD provides direct insight into growth dynamics. Space constraints in PCR gene illustrations preclude error bar inclusion. However, we'll provide detailed statistical analyses in the method part.

**Reviewer 3#**

1. The axolotls used in this study were originally obtained from the Ambystoma Genetic Stock Center (AGSC) at the University of Kentucky. The AGSC maintains a colony of axolotls and provides them to research laboratories worldwide, along with housing, breeding and care information. All experiments were performed in accordance with the guidelines of the German Animal Welfare Act. Due to these guidelines and after consultation of the veterinary inspection office as well as the “Landesamt für Lebensmittelsicherheit und Verbraucherschutz” (LAVES, relevant authority for animal trials in lower Saxony, Germany) mucus harvesting from the animals was classified as non invasive.
2. The chromatography and mass spectrometric analysis were conducted using an AXIMA Performance MALDI-TOF/TOF mass spectrometer (Shimadzu) at the Fraunhofer Institute in Hannover. For the chromatography, standard injection volumes of 5 μL were used. The obtained fractions were subsequently analyzed by liquid chromatography-mass spectrometry. Peptide identification was carried out using the instrument's integrated software, which supports fully automated proteomics experiments and LC-MALDI analyses. We have revised the manuscript to include these details. Thank you for bringing this to our attention.
3. The peptide identification process was carried out using the AXIMA Performance MALDI-TOF/TOF mass spectrometer (Shimadzu) at the Fraunhofer Institute in Hannover. For peptide identification, we used the instrument's integrated software, which supports fully automated proteomics experiments and LC-MALDI analyses. Regarding the similarity of the 3 (Peptide 1, 12,13) identified sequences to those in existing databases:

BLASTp analysis revealed no significant sequence homology for peptides 1 and 12 (E-value > 0.01). Peptide 13 showed weak alignments with AMPDB_42975|A0A2V2ABU9 (E-value = 3.7) and AMPDB_41234|A0A2A2DWC7 (E-value = 7.1), indicating statistically insignificant similarities likely occurring by chance.

4. The RPMI-1640 medium used in this study was obtained from Thermo Fisher Scientific (catalog number 11875093). The FBS was Gibco Premium (Performance Plus) FBS, catalog number 16000044, sourced from the United States.
5. The apoptosis assay was performed using the Apo-ONE® Homogeneous Caspase-3/7 Assay Kit (Promega) according to the manufacturer's instructions. While we acknowledge the reviewer's valid points, we can explain our approach as follows:
a) Negative control: The negative control (cells treated with vehicle only) was indeed part of our experiment. However, we did not include this data in Figure 2 for the following reasons:
   a. The fluorescence levels observed in the negative control were consistently low, indicating minimal background caspase-3/7 activity.
   b. The results for the negative control were similar to those observed in the untreated MCF10A cells, which serve as a non-cancerous cell line control in our study.
b) Positive control: A traditional positive control was not included in this study for the following reasons:a) The Apo-ONE® Assay Kit is designed to be highly sensitive and specific for caspase-3/7 activity, which are key indicators of apoptosis. The assay's reliability has been extensively validated by the manufacturer and in numerous published studies. The peptides themselves serve as test substances, and those showing significant increases in caspase-3/7 activity compared to the negative control can be considered positive results. The use of a traditional apoptosis inducer (e.g., staurosporine) might overshadow the more subtle effects of the peptides, potentially leading to underestimation of their apoptotic potential.

While we understand that a positive control can provide a benchmark for comparison, we believe that the relative increases in caspase-3/7 activity induced by the peptides, compared to the negative control, provide sufficient evidence of their apoptotic potential.

6. The RT2 Profiler PCR Array is a 96-well plate that contains pre-designed primer assays for a focused panel of genes related to a specific pathway or disease. It's important to note that while the specific sequences are not provided, the primers are thoroughly tested and optimized to ensure reliable and reproducible results for pathway-focused gene expression analysis. https://geneglobe.qiagen.com/us/product-groups/rt2-profiler-pcr-arrays/PAHS-131Z
7. The complete sequences of the three most effective antimicrobial peptides (AMPs) identified in this study can be found in the Supplementary (S3) Information.
8. We acknowledge that while a concentration-dependent trend was observed, the differences between concentrations were not always statistically significant for all peptides. This variability highlights the complex nature of peptide-induced apoptosis and suggests that factors beyond concentration, such as peptide sequence and cellular uptake, may influence their effectiveness. We have focused on presenting data for peptides 1, 12, and 13 as they showed the most significant and consistent effects on apoptosis induction. Peptides exhibiting inconsistent apoptotic activity and were excluded from primary figures to emphasize statistically significant results.

**Reviewer 4 #**

1. Regarding the peptide sequences: We have included the sequences of three peptides, including those that show significant antimicrobial activity, in Supplementary Table S1.
2. Concerning the mass spectrometer information: In the Materials and Methods section, we have add the following sentence:"Peptide analysis was performed using an AXIMA Performance MALDI-TOF/TOF mass spectrometer (Shimadzu) at the Fraunhofer Institute in Hannover."
3. We agree that "unknown nature" was imprecise. We've revised the sentence to:"Mass spectrometric analysis of the fractionated axolotl skin mucus revealed 4,986 peptide sequences."
